# Supplementary material for: Targeting necroptosis in muscle fibers ameliorates inflammatory myopathies
Source: Nat Commun. 2022 Jan 10;13:166. doi: 10.1038/s41467-021-27875-4 (PMC8748624; doi:10.1038/s41467-021-27875-4)
Supplement: Supplementary file 1 — Supplementary Information [file 41467_2021_27875_MOESM1_ESM.pdf]

# Supplementary Materials

## Supplementary Figures:

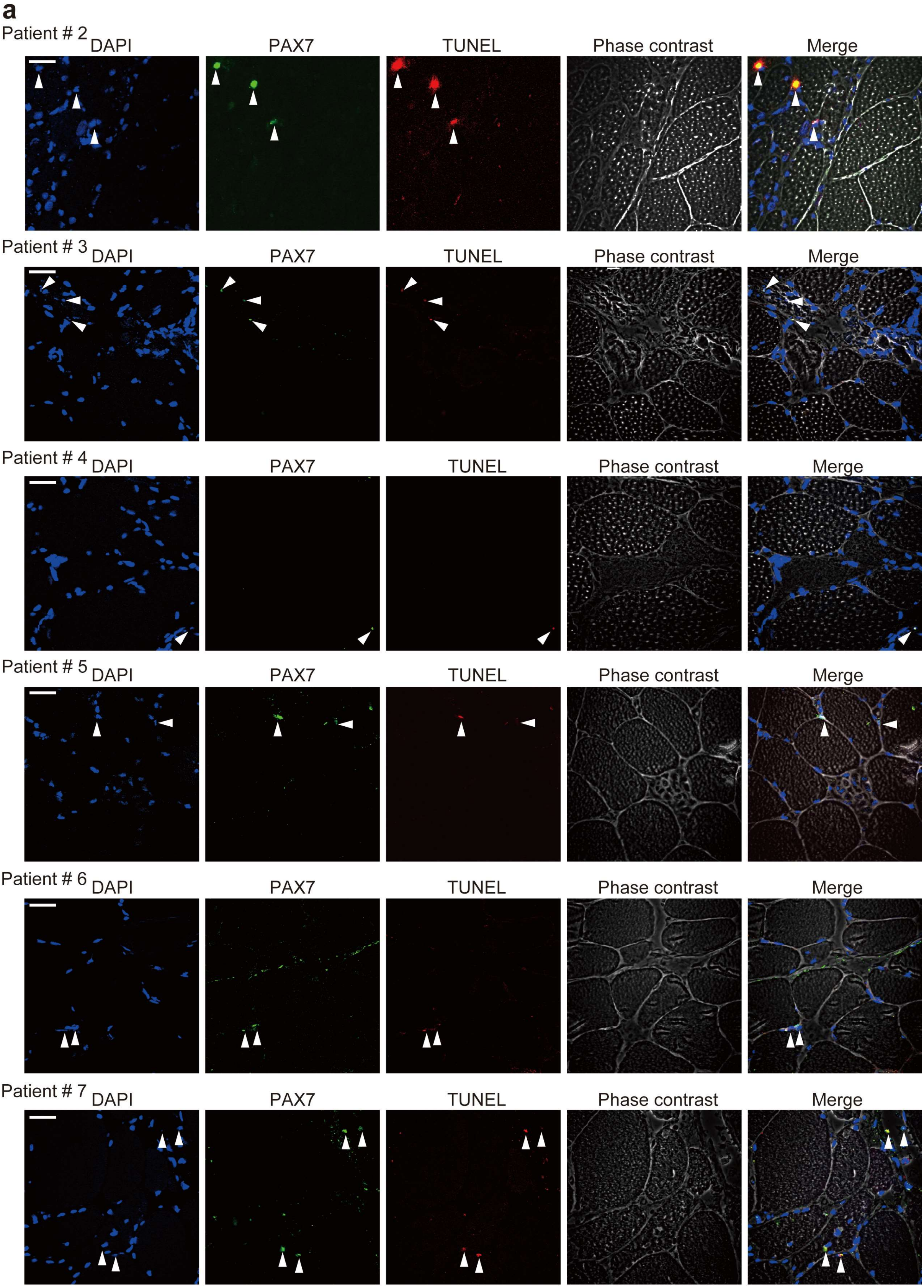

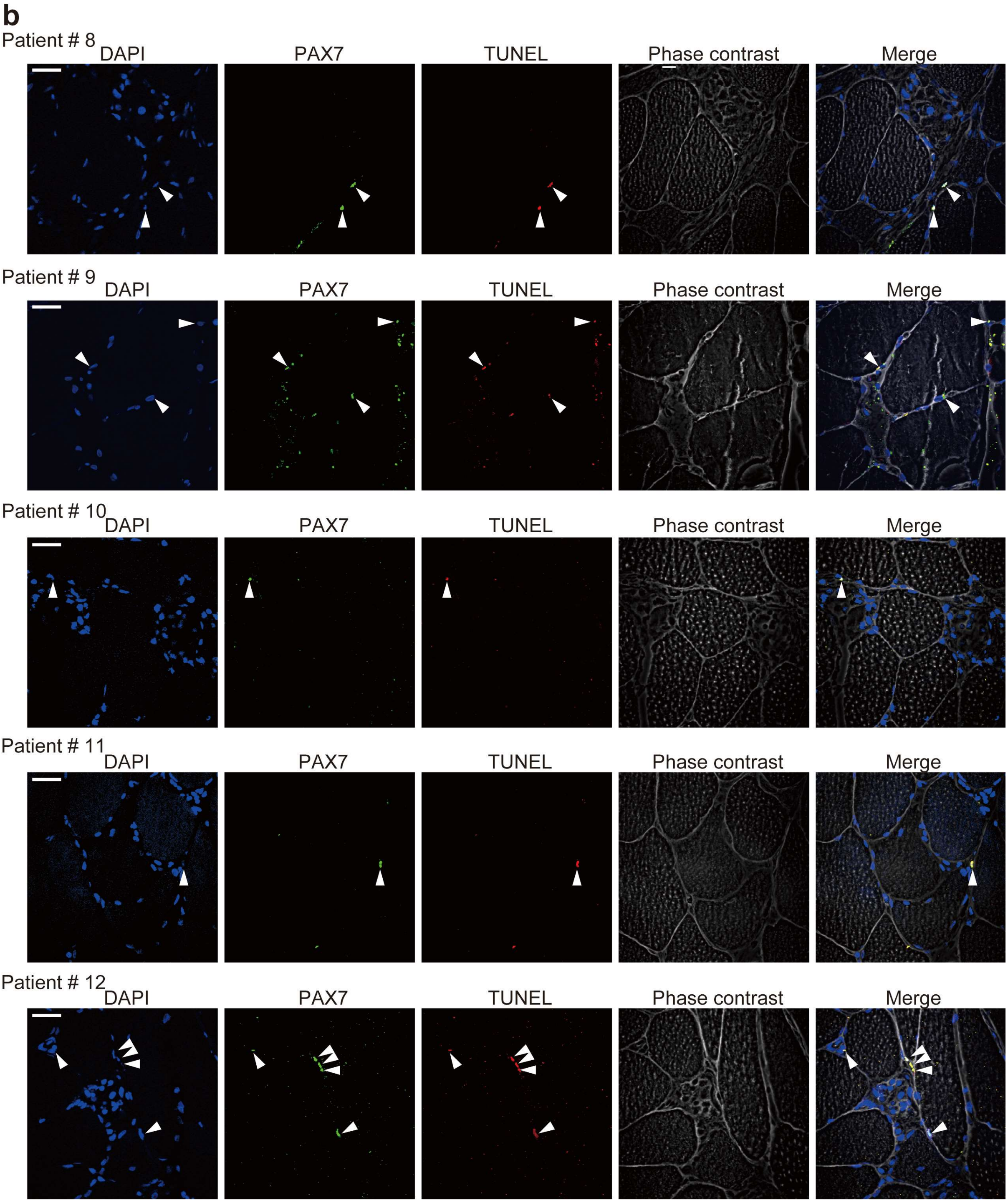
b
Patient # 8
DAPI
PAX7
TUNEL
Phase contrast
Merge
Patient # 9
DAPI
PAX7
TUNEL
Phase contrast
Merge
Patient # 10
DAPI
PAX7
TUNEL
Phase contrast
Merge
Patient # 11
DAPI
PAX7
TUNEL
Phase contrast
Merge
Patient # 12
DAPI
PAX7
TUNEL
Phase contrast
Merge

**c**

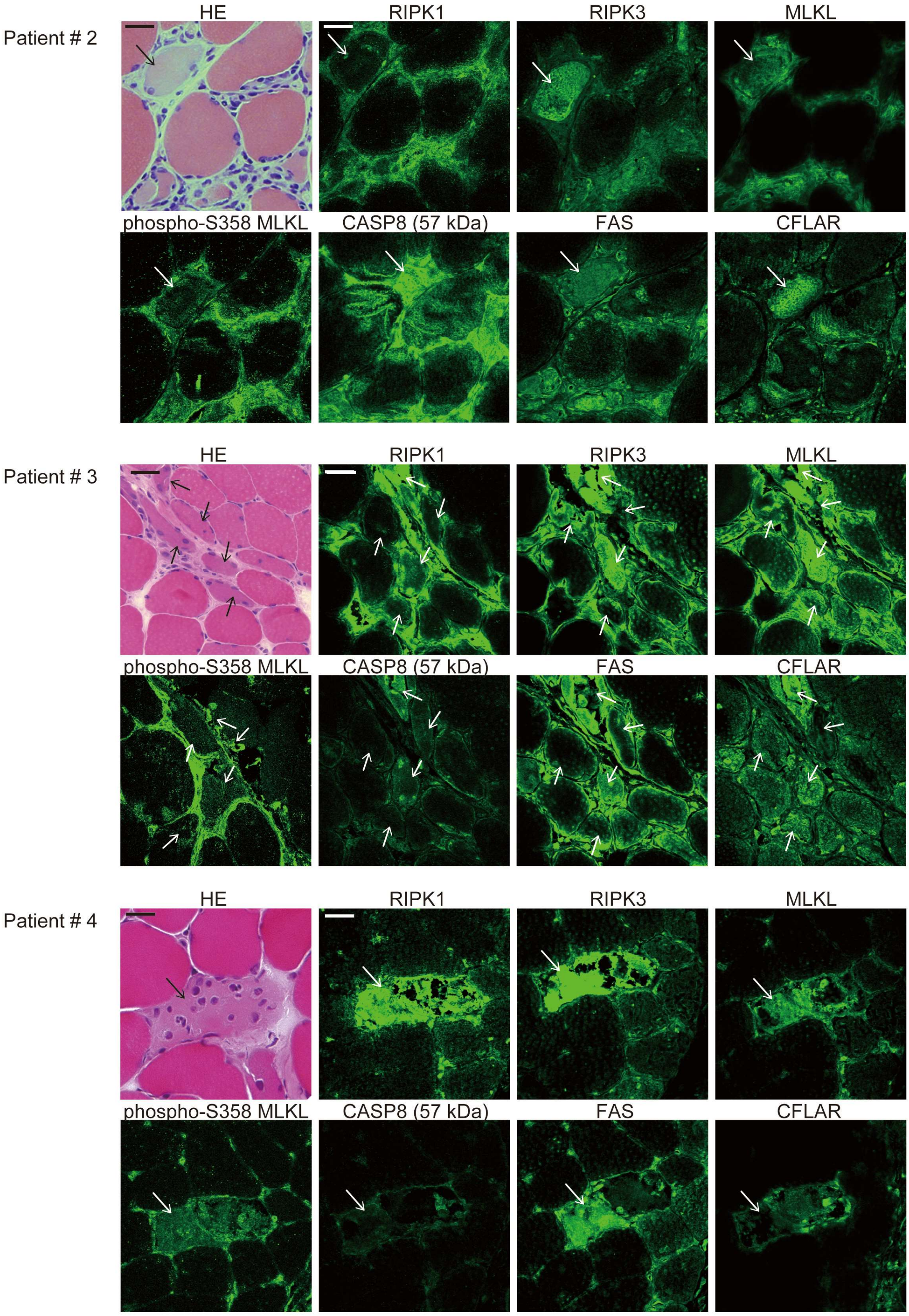
Patient # 2
HE
RIPK1
RIPK3
MLKL
phospho-S358 MLKL
CASP8 (57 kDa)
FAS
CFLAR
Patient # 3
HE
RIPK1
RIPK3
MLKL
phospho-S358 MLKL
CASP8 (57 kDa)
FAS
CFLAR
Patient # 4
HE
RIPK1
RIPK3
MLKL
phospho-S358 MLKL
CASP8 (57 kDa)
FAS
CFLAR

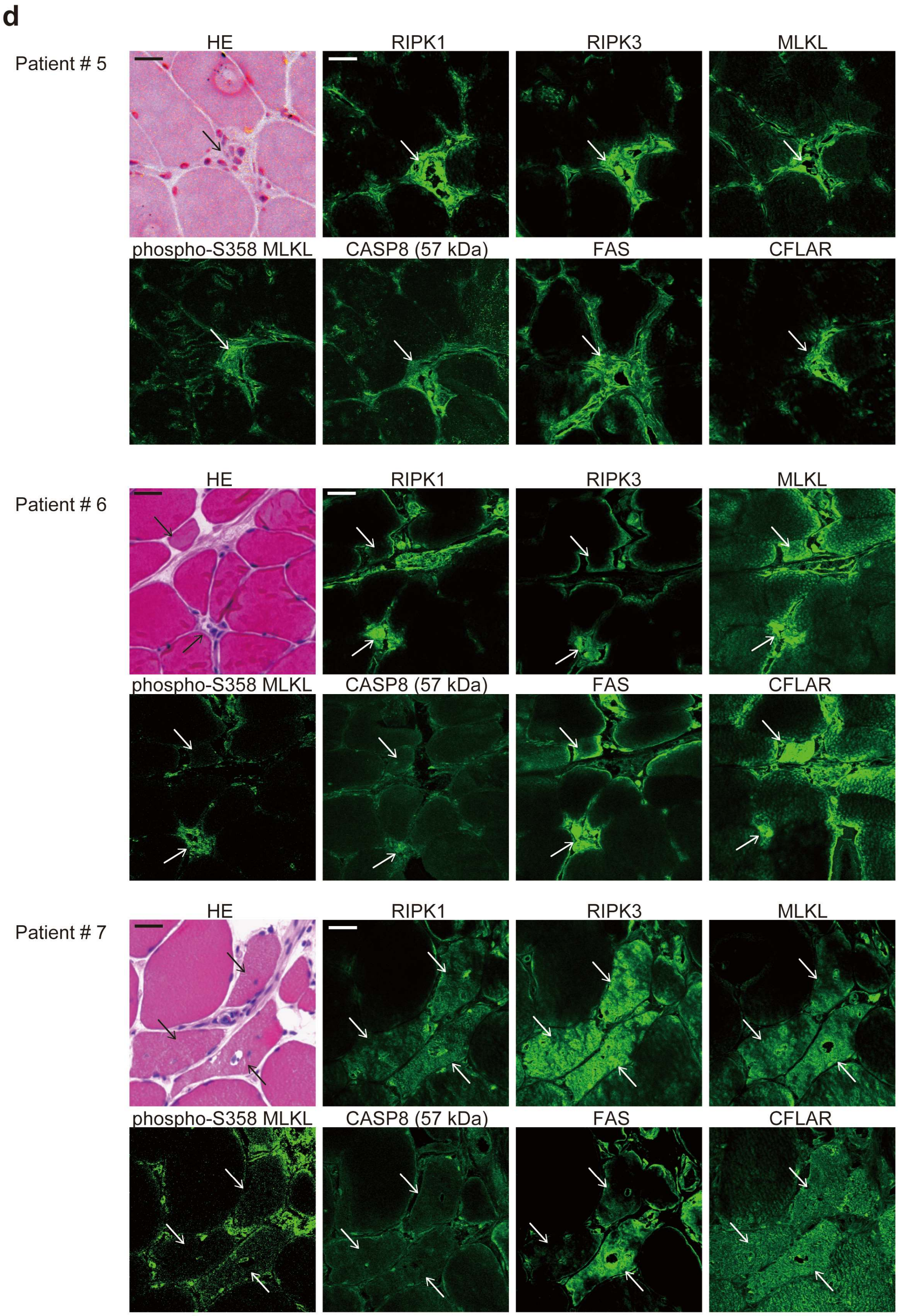
d
Patient # 5
HE
RIPK1
RIPK3
MLKL
phospho-S358 MLKL
CASP8 (57 kDa)
FAS
CFLAR
Patient # 6
HE
RIPK1
RIPK3
MLKL
phospho-S358 MLKL
CASP8 (57 kDa)
FAS
CFLAR
Patient # 7
HE
RIPK1
RIPK3
MLKL
phospho-S358 MLKL
CASP8 (57 kDa)
FAS
CFLAR

**e**

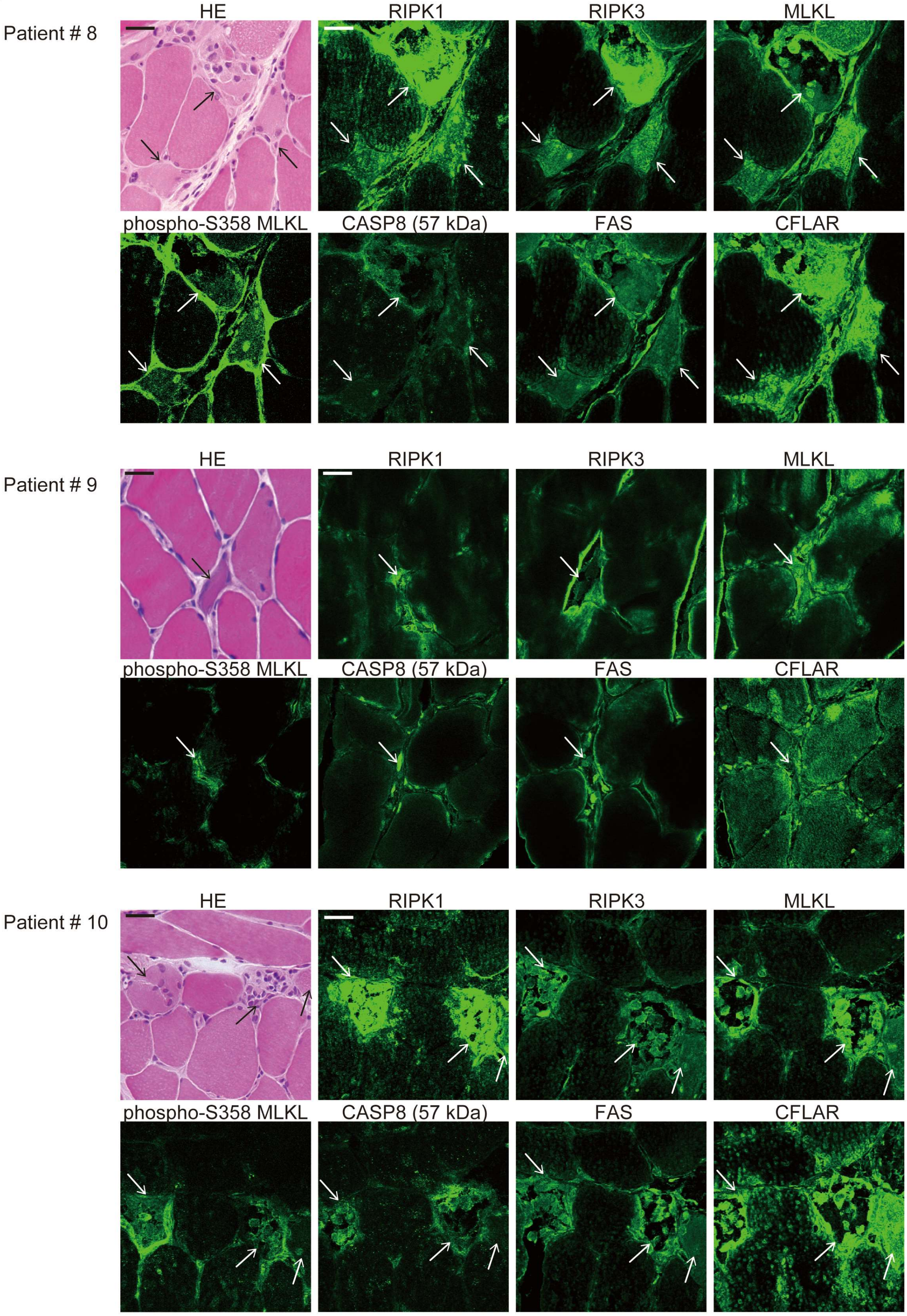

Patient # 8
HE
RIPK1
RIPK3
MLKL
phospho-S358 MLKL
CASP8 (57 kDa)
FAS
CFLAR
Patient # 9
HE
RIPK1
RIPK3
MLKL
phospho-S358 MLKL
CASP8 (57 kDa)
FAS
CFLAR
Patient # 10
HE
RIPK1
RIPK3
MLKL
phospho-S358 MLKL
CASP8 (57 kDa)
FAS
CFLAR

**f**

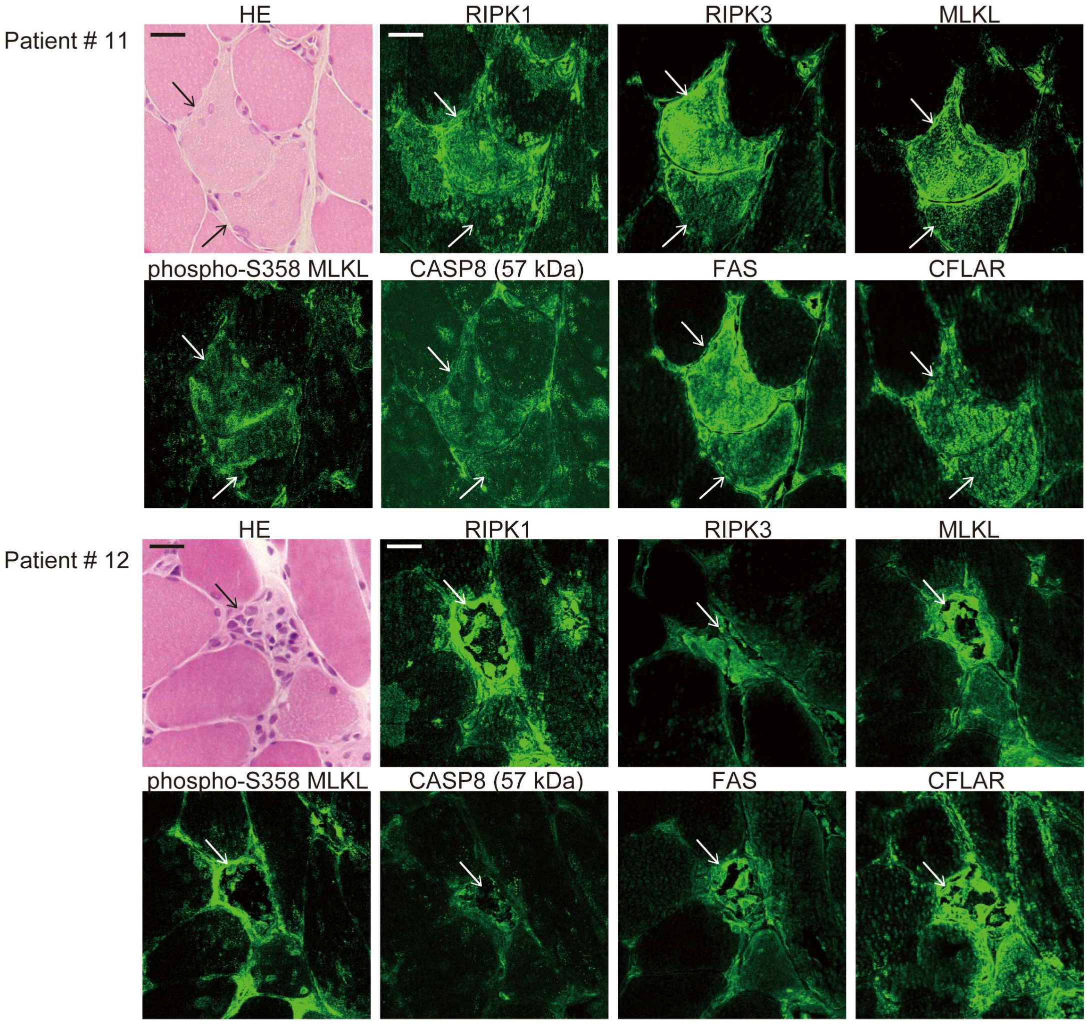
Patient # 11
HE
RIPK1
RIPK3
MLKL
phospho-S358 MLKL
CASP8 (57 kDa)
FAS
CFLAR
Patient # 12
HE
RIPK1
RIPK3
MLKL
phospho-S358 MLKL
CASP8 (57 kDa)
FAS
CFLAR

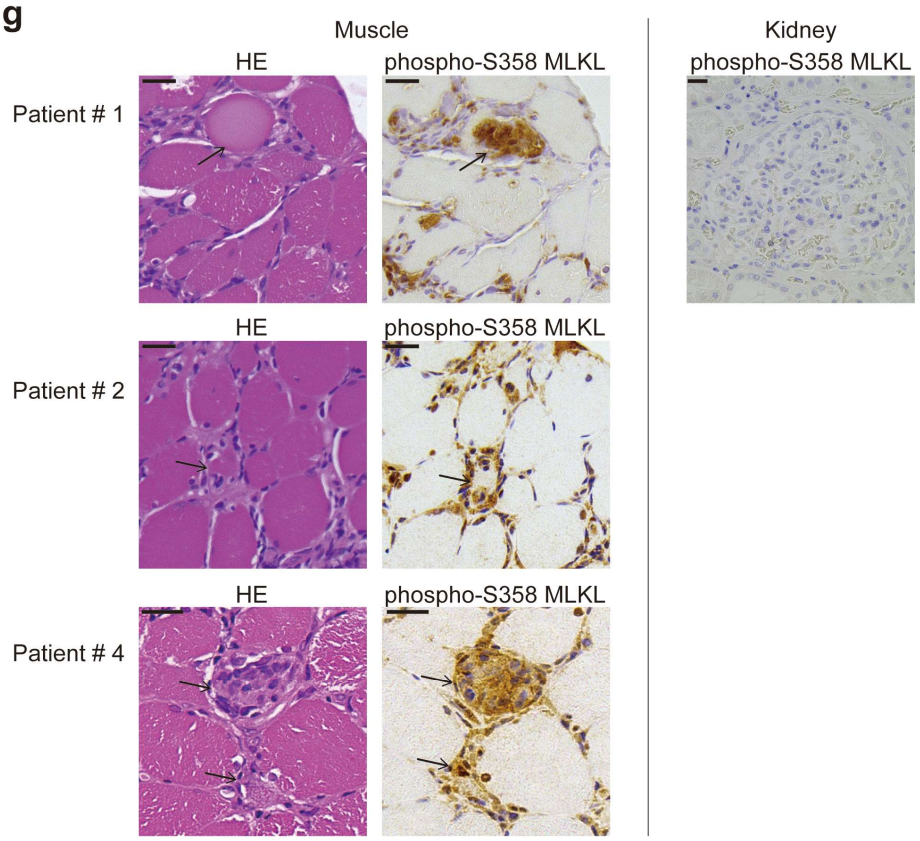
g
Muscle
Kidney
HE
phospho-S358 MLKL
phospho-S358 MLKL
Patient # 1
HE
phospho-S358 MLKL
Patient # 2
HE
phospho-S358 MLKL
Patient # 4

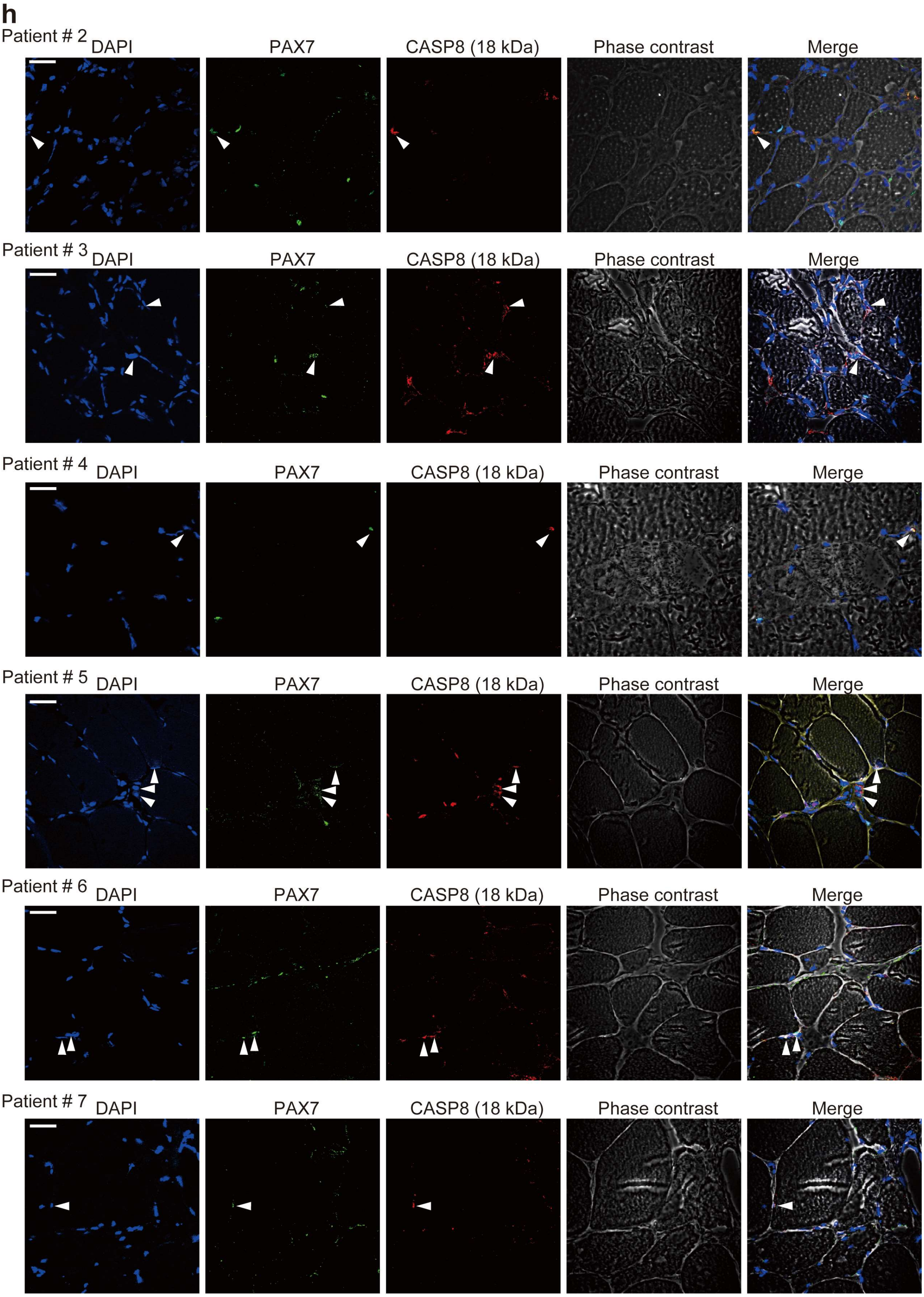
h
Patient # 2
DAPI
PAX7
CASP8 (18 kDa)
Phase contrast
Merge
Patient # 3
DAPI
PAX7
CASP8 (18 kDa)
Phase contrast
Merge
Patient # 4
DAPI
PAX7
CASP8 (18 kDa)
Phase contrast
Merge
Patient # 5
DAPI
PAX7
CASP8 (18 kDa)
Phase contrast
Merge
Patient # 6
DAPI
PAX7
CASP8 (18 kDa)
Phase contrast
Merge
Patient # 7
DAPI
PAX7
CASP8 (18 kDa)
Phase contrast
Merge

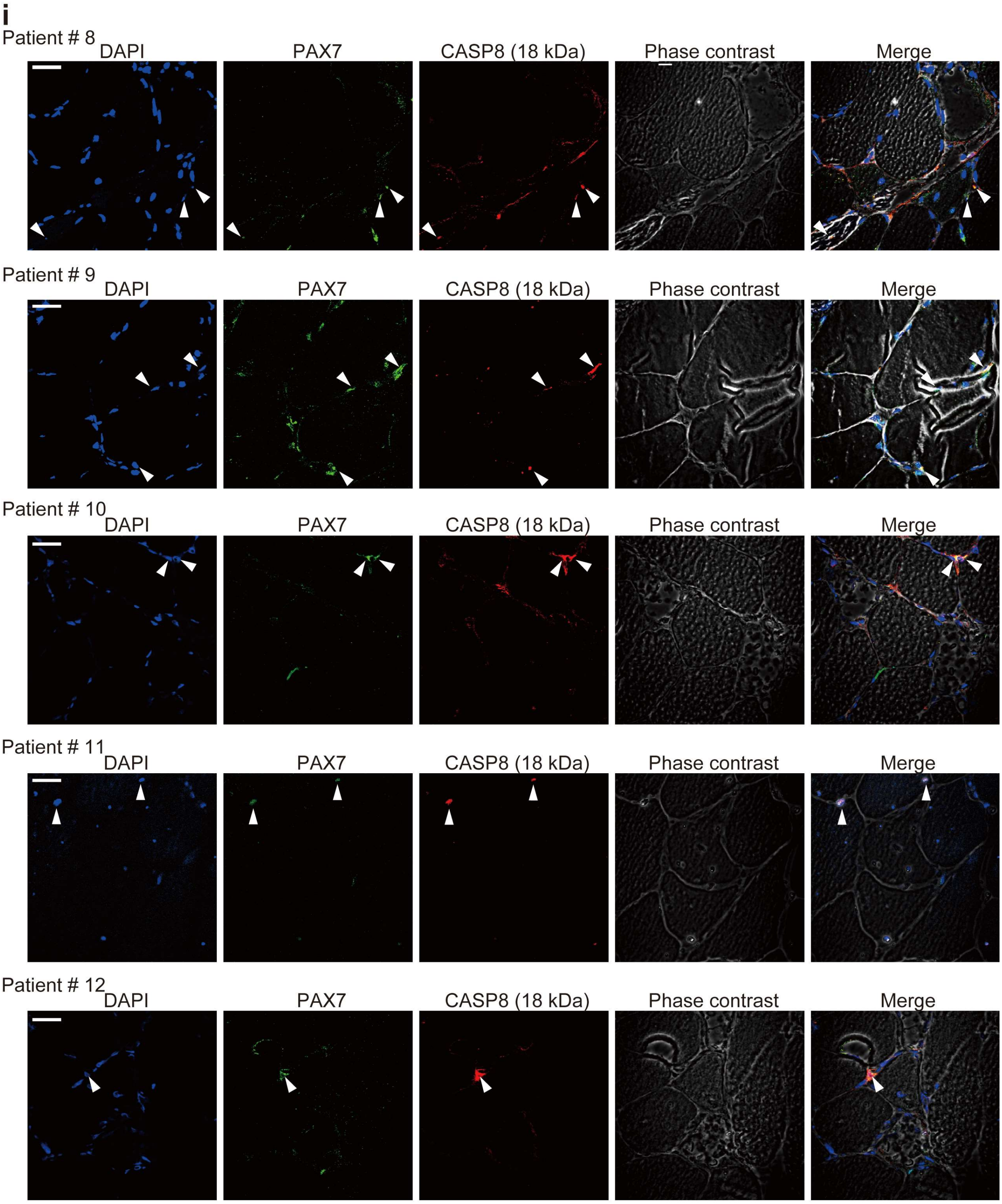


**Supplementary Figure 1. Expression of necroptosis-associated proteins in dying muscle fibers in PM and DM (additional images).** (**a-f**, **h**, and **i**) Representative images of muscle specimens of eight PM (patient #2-9) and three DM (patient #10-12) donors. Scale bars indicate 20 μm. (**a, b**) Immunofluorescence staining against PAX7 (green) and the TUNEL staining (red). Nuclei were counterstained with DAPI (blue). Arrowheads indicate TUNEL

positive PAX7 positive satellite cells. (**c-f**) HE and immunofluorescence staining against RIPK1, RIPK3, MLKL, phosphorylated MLKL at S358 (phospho-S358 MLKL), CASP8 (57 kDa), FAS, and CFLAR (green). Arrows indicate the dying muscle fibers, which showed reduced eosin staining in the cytoplasm. Nuclei were counterstained with DAPI (blue). (**g**) HE and immunohistochemical staining against phospho-S358 MLKL of formalin-fixed and paraffin-embed (FFPE) sections of muscle samples of PM (patient #1, 2, and 4) and renal samples from healthy controls. Representative images of muscle specimens of PM are shown. The arrows indicate the dying muscle fibers. The renal FFPE specimen was shown as a negative control for the expression of phosphor-S358 MLKL. Scale bars indicate 20 μm. (**h, i**) Immunofluorescence staining against PAX7 (green) and active 18 kDa CASP8 subunit (red). Nuclei were counterstained with DAPI (blue). Arrowheads indicate active 18 kDa CASP8 subunit positive PAX7 positive satellite cells. Scale bars indicate 20 μm. (**a-i**) Every histological examination was performed at least twice for each patient, and the representative images are shown.

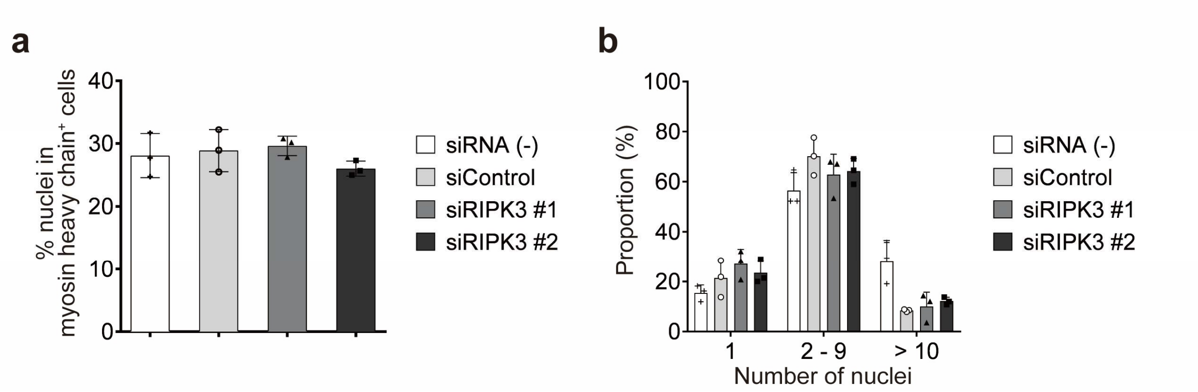


**Supplementary Figure 2. The effect of silencing Ripk3 on the differentiation to myotubes.** (**a**) The percentage of the number of nuclei within myosin heavy chain-positive myotubes out of the total number of nuclei in the microscopic fields. (**b**) The proportion of myotubes with indicated number of nuclei. (**a**, **b**) The number of evaluated cells in each well

in triplicate was as follows; siControl: n = 116, 114, 179, siRIPK3 #1: n = 149, 137, 146, siRIPK3 #2: n = 100, 139, 130. Data are presented as mean and SD of triplicate experiments.

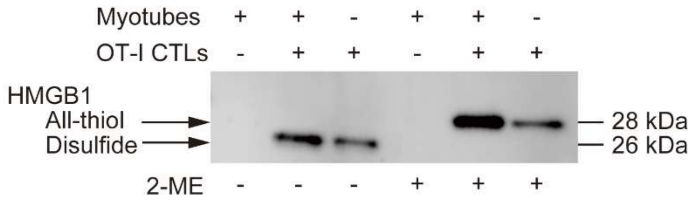


**Supplementary Figure 3. Immunoblotting of culture supernatants for analysis of redox isoforms of HMGB1.** The supernatant of the co-culture of H2K$^{b}$OVA-myotubes and OT-I CTLs or the monoculture of H2K$^{b}$OVA-myotubes or OT-I CTLs for 20 hours were heated to denature in the presence (+) or absence (-) of 2-mercaptoethanol (ME), and evaluated by immunoblotting. The data represent two independent experiments.

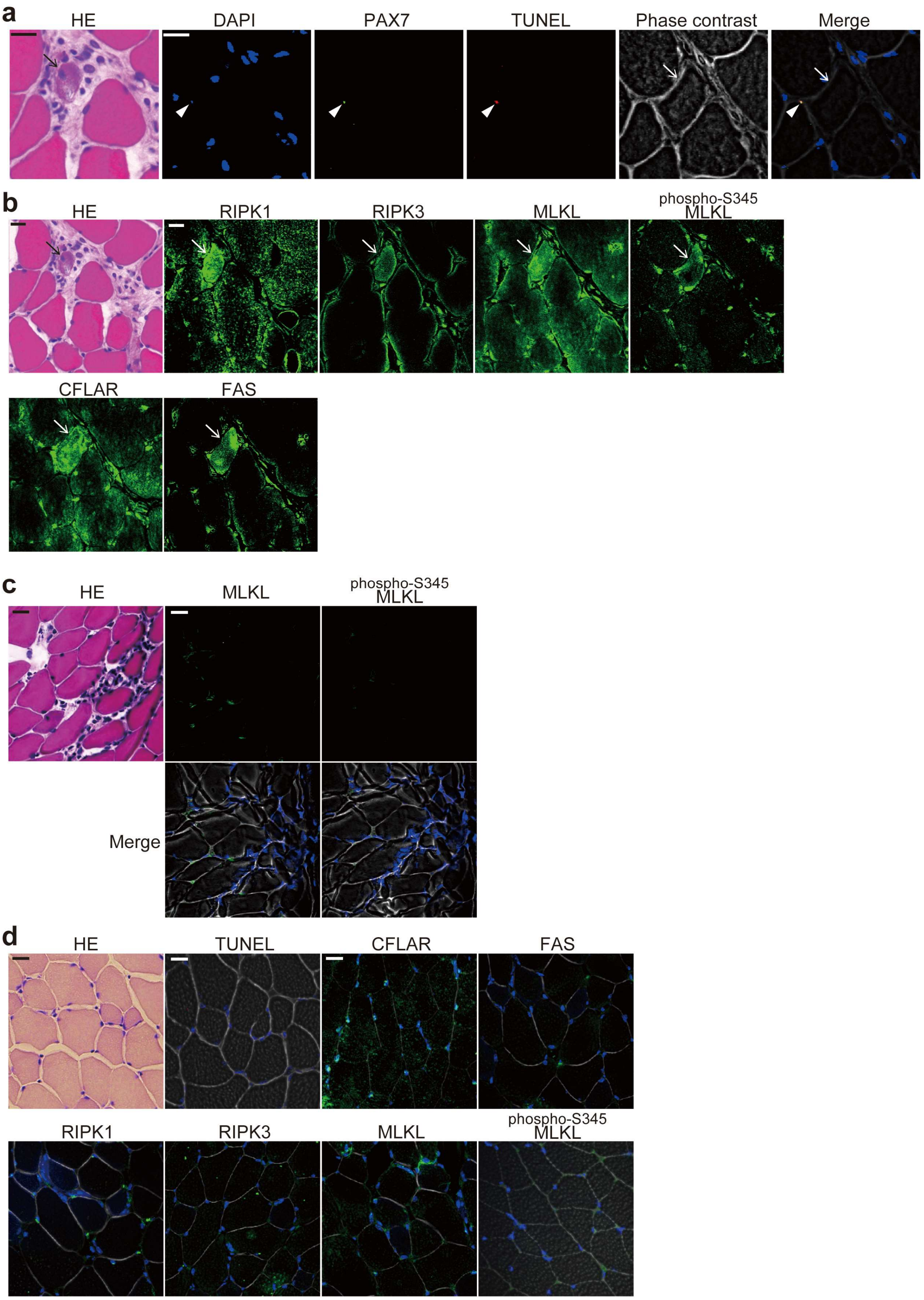


**Supplementary Figure 4. Expression of necroptosis-associated proteins in inflamed area of wild type and *Mlkl*$^{-/-}$ CIM muscle and uninflamed area of CIM muscle.** **(a)** HE,

immunofluorescence staining against PAX7 (green), and the TUNEL staining (red) in the inflamed area of wild type CIM muscle (additional images). Nuclei were counterstained with DAPI (blue) of inflamed area of wile type CIM muscle (additional images). The arrow indicates the dying muscle fiber and the arrowhead indicates PAX7 positive TUNEL positive satellite cell. Scale bars indicate 20 μm. (**b**) HE and immunofluorescence staining (green) for the expression of CFLAR, FAS, RIPK1, RIPK3, MLKL, and phosphorylated-S345 (phospho-S345) MLKL in the inflamed area of wild type CIM muscle (additional images). The arrow indicates the dying muscle fiber. Scale bars indicate 20 μm. (**c**) HE and immunofluorescence staining (green) for the expression of MLKL and phospho-S345 MLKL in the inflamed area of muscle specimens of *Mlkl*$^{-/-}$ CIM mice conducted to show the specificity of the antibodies. The merged images with nuclei (blue) and the phase contrast are also shown. Scale bars indicate 20 μm. (**d**) HE, TUNEL (red), and immunofluorescence staining (green) for the expression of CFLAR, FAS, RIPK1, RIPK3, MLKL, and phospho-S345 MLKL in the uninflamed area of muscle specimens of CIM mice. The merged images with nuclei (blue) and the phase contrast are shown in TUNEL and immunofluorescence staining. Scale bars indicate 20 μm. (**a-d**) Each histological examination was performed at least twice, and the representative images are shown.

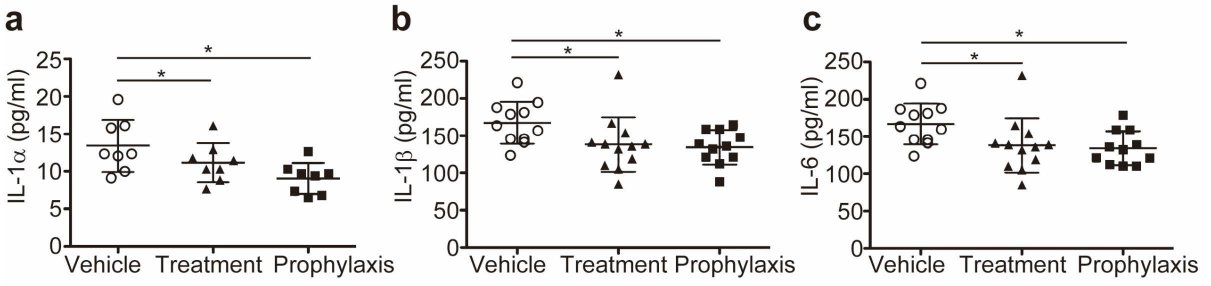


**Supplementary Figure 5. The levels of inflammatory cytokines in the muscle of CIM.** (**a-c**) The levels of IL-1α (**a**), IL-1β (**b**), and IL-6 (**c**) on day 21 in the muscles of CIM mice

treated with Nec1s or the vehicle. The treatment was started immediately after the immunization in the prophylactic group (Prophylactic Tx), or from day 7 in the therapeutic group (Therapeutic Tx). The serum of following number of mice were analyzed; (**a**) Vehicle; n = 8, Therapeutic Tx; n = 8, Prophylactic Tx; n = 8, (**b**) Vehicle; n = 11, Therapeutic Tx; n = 12, Prophylactic Tx; n = 11, and (**c**) Vehicle; n = 11, Therapeutic Tx; n = 12, Prophylactic Tx; n = 11. The data are presented as mean ± SD. One-way ANOVA test, followed by Dunnett's test. $*p < 0.05$. (**a-c**) represent two independent experiments.

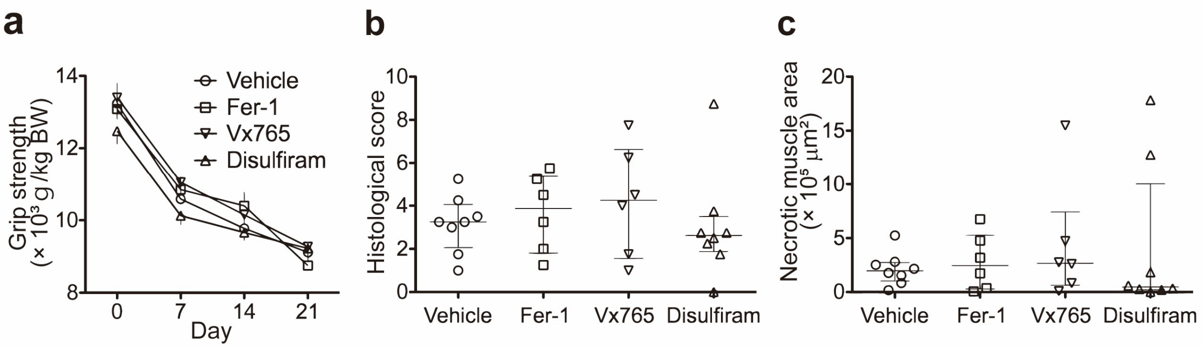


**Supplementary Figure 6. The effect of the inhibition of ferroptosis or pyroptosis in CIM.** (**a**) The grip strength of CIM mice treated with Fer-1, Vx765, Disulfiram, or vehicle. BW; Body weight of the mice. Data are presented as mean ± SD. Two-way ANOVA test, followed by Dunnett's multiple comparison test. (**b**) The histological scores of the severity of myositis on day 21 of CIM. Data are presented as median ± interquartile range. Kruskal-Wallis test, followed by Dunn's test. (**c**) The area of necrotic muscle fibers on day 21 of CIM. Data are presented as median ± interquartile range. Kruskal-Wallis test, followed by Dunn's test. (**a-c**) Vehicle; n = 8, Fer-1; n = 6, Vx765; n = 6, Disulfiram; n = 8. Data represent two independent experiments for each inhibitor.

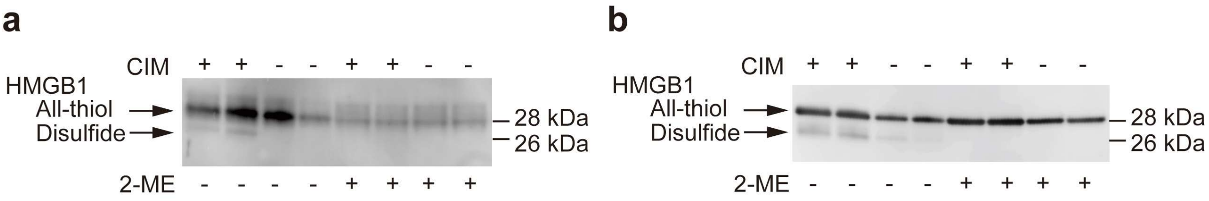


**Supplementary Figure 7. Immunoblotting of immunoprecipitated HMGB1 from muscle homogenates and serum of CIM mice for analysis of redox isoforms of HMGB1.** The muscle homogenates (**a**) and the serum (**b**) of CIM mice (CIM +) or the mice without CIM (CIM -) were immunoprecipitated with anti-HMGB1. The immunoprecipitates were heated to denature in the presence (+) or absence (-) of 2-mercaptoethanol (ME) and evaluated with immunoblotting. The data represents three independent experiments.

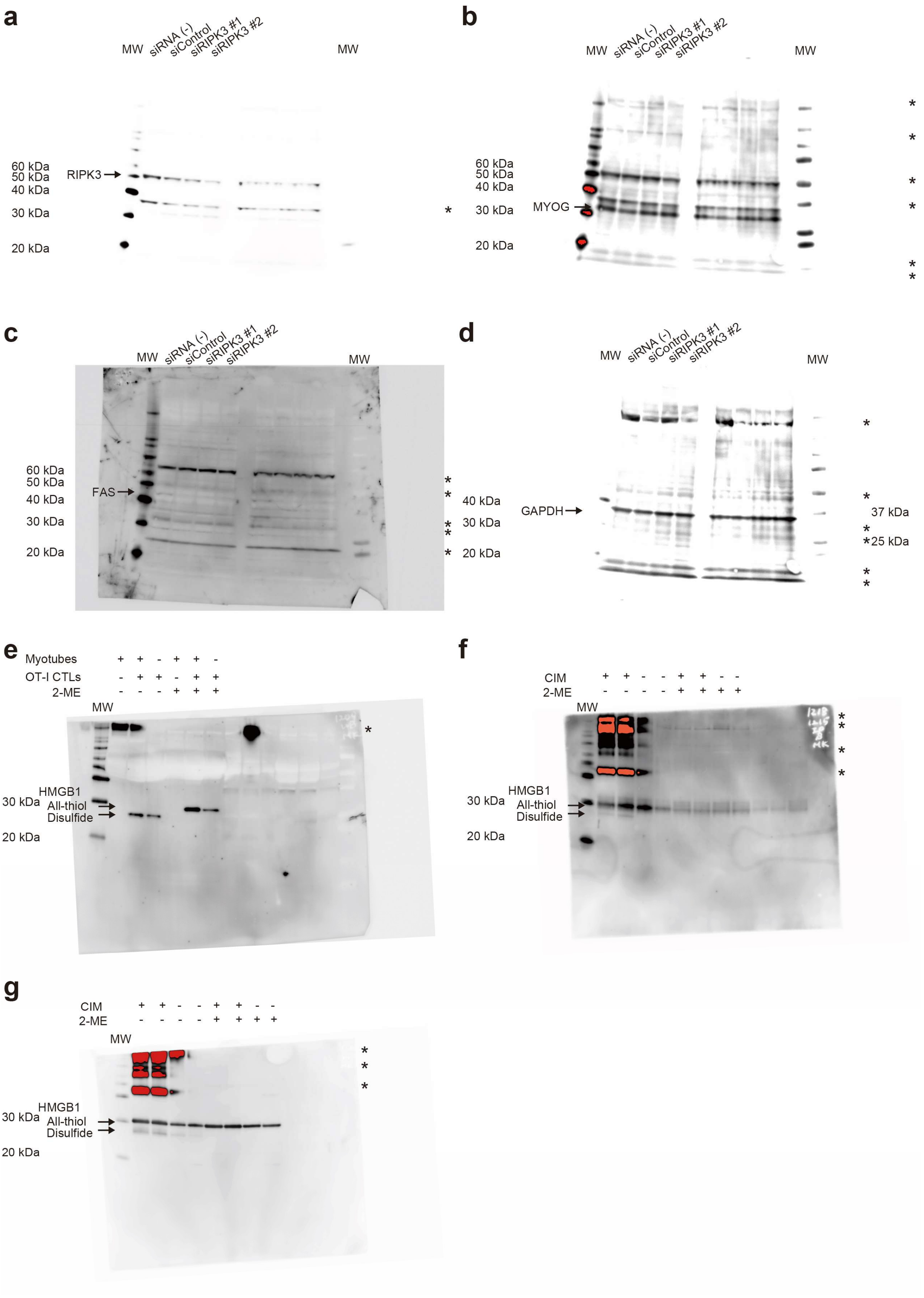


**Supplementary Figure 8. Uncropped scans of blots.** Uncropped scans corresponding to Figure 4f (**a-d**), Supplementary Figure 3 (**e**), Supplementary Figure 7a (**f**), and

Supplementary Figure 7b (**g**). Unspecific bands are indicated by *. MW, molecular weight marker.
