# Supplementary material for: Targeting necroptosis in muscle fibers ameliorates inflammatory myopathies
Source: Nat Commun. 2022 Jan 10;13:166. doi: 10.1038/s41467-021-27875-4 (PMC8748624; doi:10.1038/s41467-021-27875-4)
Supplement: Supplementary file 3 — Description of Additional Supplementary Files [file 41467_2021_27875_MOESM3_ESM.pdf]

Description of Additional Supplementary Files

Supplementary Data 1.

The clinical, serological, and histopathological features of the patients. Bohan and Peter, Bohan and Peter criteria; PM, polymyositis; DM, dermatomyositis; 2017 EULAR/ACR, 2017 European League Against Rheumatism/American College of Rheumatology (EULAR/ACR) classification criteria for adult and juvenile idiopathic inflammatory myopathies; CADM, clinically amyopathic DM; MMT, manual muscle testing; IP, interstitial pneumonia; CK, creatinine kinase (reference interval, male: 62-287 U/L; female: 45-163 U/L); ANA, antinuclear antibodies; MSA, myositis specific antibodies; ARS, anti-aminoacyl tRNA synthetase antibodies; Jo-1, anti-Jo-1 antibodies; TIF1[illegible], anti-transcription intermediary factor 1-gamma antibodies; SS-A, anti–Sjögren's-syndrome-related antigen A autoantibodies; SS-B, anti–Sjögren's-syndrome-related antigen B autoantibodies; RNP, anti-ribonucleoprotein antibodies; AMA2, anti-mitochondrial M2 antibodies; ACA, anti-centromere antibodies; EMG, electromyography; NA, not analyzed; MAC, membrane attack complex. The characters in the EMG findings indicate as follows; (a) short, small, low-amplitude polyphasic motor unit potentials, (b) fibrillation potentials at rest, and (c) bizarre high-frequency repetitive discharges.
