# Supplementary material for: Targeting necroptosis in muscle fibers ameliorates inflammatory myopathies
Source: Nat Commun. 2022 Jan 10;13:166. doi: 10.1038/s41467-021-27875-4 (PMC8748624; doi:10.1038/s41467-021-27875-4)
Supplement: Supplementary file 5 — Reporting Summary [file 41467_2021_27875_MOESM5_ESM.pdf]

nature research

Corresponding author(s): Shinsuke Yasuda
Last updated by author(s): Oct 26, 2021

Reporting Summary

Nature Research wishes to improve the reproducibility of the work that we publish. This form provides structure for consistency and transparency in reporting. For further information on Nature Research policies, see our Editorial Policies and the Editorial Policy Checklist.

Statistics

For all statistical analyses, confirm that the following items are present in the figure legend, table legend, main text, or Methods section.

n/a	Confirmed	
☐	☒	The exact sample size (*n*) for each experimental group/condition, given as a discrete number and unit of measurement
☐	☒	A statement on whether measurements were taken from distinct samples or whether the same sample was measured repeatedly
☐	☒	The statistical test(s) used AND whether they are one- or two-sided<br>*Only common tests should be described solely by name; describe more complex techniques in the Methods section.*
☐	☒	A description of all covariates tested
☐	☒	A description of any assumptions or corrections, such as tests of normality and adjustment for multiple comparisons
☐	☒	A full description of the statistical parameters including central tendency (e.g. means) or other basic estimates (e.g. regression coefficient) AND variation (e.g. standard deviation) or associated estimates of uncertainty (e.g. confidence intervals)
☐	☒	For null hypothesis testing, the test statistic (e.g. *F*, *t*, *r*) with confidence intervals, effect sizes, degrees of freedom and *P* value noted<br>*Give P values as exact values whenever suitable.*
☒	☐	For Bayesian analysis, information on the choice of priors and Markov chain Monte Carlo settings
☒	☐	For hierarchical and complex designs, identification of the appropriate level for tests and full reporting of outcomes
☐	☒	Estimates of effect sizes (e.g. Cohen's *d*, Pearson's *r*), indicating how they were calculated

Our web collection on statistics for biologists contains articles on many of the points above.

Software and code

Policy information about availability of computer code

Data collection	In the in vitro study and analysis of immunofluorescence staining, images were taken with FV-10 DOC (Olympus, Japan). For western blotting, the images were taken with LAS-3000 Imaging System (Fujifilm, Japan).
Data analysis	For the analysis of images including in vitro study and immunofluorescence staining, FV10-ASW (Ver.4.2a) and ImageJ (Ver.1.52u) softwares were used. For the statistical analysis, PRISM (Ver.6, 7, and 8, GraphPad, CA, USA) was used.

For manuscripts utilizing custom algorithms or software that are central to the research but not yet described in published literature, software must be made available to editors and reviewers. We strongly encourage code deposition in a community repository (e.g. GitHub). See the Nature Research guidelines for submitting code & software for further information.

Data

Policy information about availability of data

All manuscripts must include a data availability statement. This statement should provide the following information, where applicable:
- Accession codes, unique identifiers, or web links for publicly available datasets
- A list of figures that have associated raw data
- A description of any restrictions on data availability

Source data are provided with this paper. All of the data are available from the corresponding author upon reasonable request.

Field-specific reporting

Please select the one below that is the best fit for your research. If you are not sure, read the appropriate sections before making your selection.

☒ Life sciences ☐ Behavioural & social sciences ☐ Ecological, evolutionary & environmental sciences

For a reference copy of the document with all sections, see nature.com/documents/nr-reporting-summary-flat.pdf

Life sciences study design

All studies must disclose on these points even when the disclosure is negative.

Sample size

For in vitro studies, the number of cells to analyzed was not determined using a statistical method, but was determined so that as many cells as possible could be observed to the extent that cell death of each cell could be discerned (Kamiya M., et al. Rheumatol. 59, 224-232 (2020)), as described in the methods section of the manuscript: "All of the myoblasts or myotubes in five to eight randomly taken microscopic fields which contain about 5-10 myoblasts or 5-20 myotubes per field were evaluated". For the in vivo study, the number of mice per group was not determined using a statistical method, but selected conventionally taking into account the variability of the parameters including histological scores (Sugihara T., et al. Arthritis Rheum. 62, 3088-3092 (2010), Yoshihashi-Nakazato Y., et al. Arthritis Rheum. 68, 1505-1510 (2016)). For the histological analysis on human samples, the number of the patients was not determined using a statistical method, but was selected conventionally to be sufficient to demonstrate their histological characteristics (Dorph C., et al. Ann Rheum Dis. 65, 1565-1571 (2006)).

Data exclusions

No data exclusions were made.

Replication

All experiments were replicated independently at least twice and the exact numbers of replication were described in the legend of the corresponding figures. All attempts at replication were successful and provided similar results.

Randomization

In the in vitro experiments, we have confirmed that there were no differences in the culture conditions and the results of cell death assays due to the different locations of wells in a chamber slides or a plate. For every in vitro experiments, the cells that had been cultured and passaged in the same culture dish were seeded evenly into the each well in the same chamber slides or a culture plate and cultured or differentiated in the same manner until the cytotoxic assay. The locations of the wells for cells in each condition were assigned to predetermined alignment so as to reduce the chance of mishandling. In all mice experiments, mice were allocated to each treatment groups in a random manner.

Blinding

The investigators were blinded to group allocation during data collection and analysis in all experiments.

Reporting for specific materials, systems and methods

We require information from authors about some types of materials, experimental systems and methods used in many studies. Here, indicate whether each material, system or method listed is relevant to your study. If you are not sure if a list item applies to your research, read the appropriate section before selecting a response.

Materials & experimental systems

n/a	Involved in the study
☐	☒ Antibodies
☐	☒ Eukaryotic cell lines
☒	☐ Palaeontology and archaeology
☐	☒ Animals and other organisms
☐	☒ Human research participants
☒	☐ Clinical data
☒	☐ Dual use research of concern

Methods

n/a	Involved in the study
☒	☐ ChIP-seq
☒	☐ Flow cytometry
☒	☐ MRI-based neuroimaging

Antibodies

Antibodies used

The following antibodies and reagents were used for immunofluorescence staining: anti-PAX7 (PAX7497, Abcam, Cambridge, UK, 1:200), anti-RIPK1 (rabbit polyclonal, NOVUS Biologicals, Litleton, Colorado, USA, #NBP1-77077, 1:200), anti-RIPK3 (rabbit polyclonal, Abcam, Cambridge, UK, #ab152130, 1:100), anti-MLKL (rabbit polyclonal, LSBio, Seattle, Washington, USA, #LS-C334151, 1:100), anti-MLKL (phospho S358, EPR9514, Abcam, Cambridge, UK, 1:250), anti-MLKL (phospho S345, EPR9515(2), Abcam, Cambridge, UK, 1:100), anti-CASP8 (Full length, rabbit polyclonal, Abcam, Cambridge, UK, #ab4052, 1:100), anti-CASP8 (active form p18 subunit, 2B12.1, Merck, Kenilworth, New Jersey, USA, 1:250), anti-FAS (rabbit polyclonal, Abcam, Cambridge, UK, #ab82419, 1:100), anti-CFLAR (rabbit polyclonal, Abcam, Cambridge, UK, #ab8421, 1:100), anti-HMGB1 (rabbit polyclonal, Abcam, Cambridge, UK, #ab18256, 1:500), polyclonal rabbit IgG (Abcam, Cambridge, UK, #ab37415), monoclonal rabbit IgG (EPR25A, Abcam, Cambridge, UK), monoclonal mouse IgM (GC323, Merck, Kenilworth, New Jersey, USA), anti-mouse IgG-Alexa Fluor 647 (Thermo Fisher Scientific, Waltham, Massachusetts, USA, #A21237), anti-rabbit IgG-Alexa Fluor 647 (Thermo Fisher Scientific, Waltham, Massachusetts, USA, #A21245), anti-mouse IgM-Alexa Fluor 594 (Thermo Fisher Scientific, Waltham, Massachusetts, USA, #A21044) antibodies, and Fluoromount-G Mounting Medium with DAPI (Thermo Fisher Scientific, Waltham, Massachusetts, USA). For the

immunohistochemical staining, anti-MLKL (phospho S358, EPR9514, Abcam, Cambridge, UK, 1:250), anti-CD8 (C8/144B, Nichirei, Tokyo, Japan, 1:200), anti-CD4 (1F6, Nichirei, Tokyo, Japan, 1:100), anti-CD68 (PG-M1, Dako Cytomation, Glostrup, Denmark, 1:100), anti-CD20cy (L26, Dako Cytomation, Glostrup, Denmark, 1:100), anti-C5b-9 (aE11, Dako Cytomation, Glostrup, Denmark, 1:100), anti-HLA-ABC (W6/32, Dako Cytomation, Glostrup, Denmark, 1:500), monoclonal rabbit IgG (EPR25A, Abcam, Cambridge, UK), mouse IgG1 (MAB002 R&D Systems, Minneapolis, Minnesota, USA), peroxidase-labeled amino acid polymer-conjugated goat anti-rabbit IgG (Dako Cytomation, Glostrup, Denmark), anti-mouse IgG (Dako Cytomation, Glostrup, Denmark), and diaminobenzene (Dako Cytomation, Glostrup, Denmark). For the detection of apoptosis in human and mouse muscle specimens and cultured cells, Click-iT TUNEL Alexa Fluor 594 Imaging Assay kit (Invitrogen, Carlsbad, California, USA) was used. For the time-lapse imaging, the following reagents were used: CellTrackerTM Green (Invitrogen, Carlsbad, California, USA), benzyloxycarbonyl-Val-Ala-Asp-fluoromethylketone (z-VAD-fmk, BACHEM, Bubendorf, Switzerland), necrostatin-1s (Nec1s, Haoyuan ChemExpress Co., Ltd., Shanghai, China), recombinant mouse FAS-Fc chimera protein (R&D Systems, Minneapolis, Minnesota, USA), recombinant human Fc protein (R&D Systems, Minneapolis, Minnesota, USA), Hoechst 33342 (Thermo Fisher Scientific, Waltham, Massachusetts, USA), propidium iodide (PI; Invitrogen, Carlsbad, California, USA), and FITC-conjugated Annexin V (BioLegend, San Diego, Californina, USA). For the western blotting, the following regents were used: anti-RIPK3 (rabbit polyclonal, Abcam, Cambridge, UK, #ab152130, 1:1000), anti-myogenin (EPR4789, Abcam, Cambridge, UK, 1:1000), anti- FAS (rabbit polyclonal, Abcam, Cambridge, UK, #ab82419, 1:1000), anti-GAPDH (D4C6R, Cell Signaling Technology, Danvers, Massachusetts, USA, 1:1000), anti-HMGB1 (rabbit polyclonal, Abcam, Cambridge, UK, #ab18256, 1:1000), horseradish peroxidase (HRP)-conjugated goat anti-rabbit IgG, goat anti-mouse IgG antibodies (Cell Signaling Technology, Danvers, Massachusetts, USA), and Clean-Blot IP detection Reagent (Thermo Fisher Scientific, Waltham, Massachusetts, USA). For the treatment of CIM, Anti-HMGB1 antibodies (#10-22, produced by Okayama University, Okayama, Japan) were used.

Validation

All commercially available antibodies were validated for immunohistological staining by positive control tissue staining of mice or human samples and were also validated for western blotting by using positive control cell lysates of human or mice cell lines, which were shown on the corresponding manufacturer's websites. All antibodies were well cited (https://www.citeab.com/) and also were validated in house. For the immunofluorescence staining against MLKL and phosphorrylated-S345 MLKL on CIM muscles, we used the muscles of Mlkl-/- mice as negative control.
Anti-HMGB1 antibodies (#10-22) were validated in the published manuscripts including "Liu K. et al. FASEB J 2007 (Ref 28)".

Eukaryotic cell lines

Policy information about cell lines

Cell line source(s)

C2C12 mouse myoblasts were purchased from the ATCC (ATCC #CRL-1722).

Authentication

The identity was not authenticated by our hands. We confirmed that the cells were able to be differentiated to polynuclear myotubes and expressed muscle specific proteins including myosin heavy chain and myogenin.

Mycoplasma contamination

The cell line was negative for mycoplasma contamination.

Commonly misidentified lines (See ICLAC register)

Nothing was listed as commonly misidentified lines in ICLAC registry.

Animals and other organisms

Policy information about studies involving animals; ARRIVE guidelines recommended for reporting animal research

Laboratory animals

OVA-specific class I restricted T cell receptor (TCR) transgenic mice (OT-I) and C57BL/6 mice were purchased from Charles River Japan (Kanagawa, Japan). C57BL/6 Ripk3-/- mice and C57BL/6 Mlkl-/- mice were described previously (see the reference 64 and 65). Mice were bred at a standard temperature (21 ± 1℃) under a standard 12 h light: dark cycle in a humidity-controlled environment and had ad libitum access to water and food (CE-2, CLEA). The experimental procedures were carried out at the center for animal research in Tokyo Medical and Dental University (TMDU) and Walter and Eliza Hall Institute of Medical Research (WEHI). Female C57BL/6 mice at the age of 8 weeks and female and male C57BL/6 Ripk3-/- mice, C57BL/6 Mlkl-/- mice, or their littermates at the age of 6-10 weeks were used for CIM experiments.

Wild animals

This study did not involve wild animals.

Field-collected samples

This study did not involve samples collected from the field.

Ethics oversight

All animal experiments were approved by the Institutional Animal Care and Use Committee of TMDU and Animal Ethics Committee in WEHI and were performed in accordance with the guidelines of both institutes and both countries.

Note that full information on the approval of the study protocol must also be provided in the manuscript.

Human research participants

Policy information about studies involving human research participants

Population characteristics

Muscle specimens analyzed in this study were obtained from the patients with PM (n = 9) or DM (n = 3) who met the Bohan and Peter criteria and 2017 European League Against Rheumatism/American College of Rheumatology (EULAR/ACR) classification criteria for adult and juvenile idiopathic inflammatory myopathies at Department of Rheumatology, Tokyo Medical and Dental University (TMDU) between October 2016 and April 2020. The median (interquartile range) age of the patients was 35 (34, 61) and 50 (25, 59) in PM and DM patients, respectively. Five and one female patients were included in PM and DM patients, respectively. All the patients included in the analysis showed necrotic muscle fibers but did not have the characteristic feature of immune-mediated necrotizing myopathy. The patients who were suspicious of cancer-

	associated, viral, or immune check point inhibitor-associated myositis were excluded. The clinical, serological, and histopathological features of the patients were shown in Supplementary Table 1.
Recruitment	Muscle specimens were obtained from the patients with PM (n = 9) or DM (n = 3) who met the Bohan and Peter criteria and 2017 European League Against Rheumatism/American College of Rheumatology (EULAR/ACR) classification criteria for adult and juvenile idiopathic inflammatory myopathies at Department of Rheumatology, Tokyo Medical and Dental University (TMDU) between October 2016 and April 2020. All of the patients were suspected of suffering PM/DM in routine clinical practice and histological analysis was performed for clinical necessity. We therefore consider that there was no potential self-selection bias existed in our study. Since inflammatory myopathies assumingly consist of heterogeneous subsets of disease, we included as many patients as possible to minimize the selection bias.
Ethics oversight	The study protocols were approved by the institutional review board at TMDU and are in accordance with the principles of the Declaration of Helsinki. Written informed consent was obtained from all participants.

Note that full information on the approval of the study protocol must also be provided in the manuscript.
